# Performance Evaluation of Asphalt Rubber Mixture with Additives

**DOI:** 10.3390/ma12081200

**Published:** 2019-04-12

**Authors:** Xianpeng Cheng, Yamin Liu, Wanyan Ren, Ke Huang

**Affiliations:** Highway and Airport Pavement Research Center, School of highway, Chang’an University, Xi’an 710064, China; cheng_xp@chd.edu.cn (X.C.); renwanyan@126.com (W.R.); 2017221128@chd.edu.cn (K.H.)

**Keywords:** asphalt rubber, high-temperature performance, fatigue resistance

## Abstract

Crumb rubber, as a recycled material used in asphalt mixture, has gained more attention in recent years due to environmental benefits and the advantages of its pavement, such as excellent resistance to cracking, improved durability, less road maintenance, lower road noise, etc. However, the high-temperature performance of mixture with crumb rubber does not perform well. In order to improve the performance, this paper examined the effect of additives on the laboratory performance of asphalt rubber Stone Matrix Asphalt (AR-SMA) with additives. Three groups of AR-SMA: no additives, Styrene–Butadiene–Styrene (SBS) and Granular Polymer Durable additive (GPDa) were included, with no additives as a control group. Each group was investigated at three asphalt rubber content (ARC): 6.4%, 6.9%, 7.4% with regard to high-temperature and fatigue properties. The results show that with increasing ARC, the high-temperature performance of mixture without additive decreases, and the high-temperature performance increases first and then decreases for SBS and GPDa. Moreover, the rutting resistance of AR-SMA with GPDa at 6.9% ARC performs best. Under the condition of mixtures with appropriate ARC, AR-SMA with GPDa has higher fatigue life and sensitivity to fatigue cracking than the control group. Simultaneously, the fatigue performance of AR-SMA with GPDa is not as significant as that without additive with increasing ARC. In a word, GPDa is a good choice to improve the performance of AR-SMA. However, it should be noted that optimal asphalt content of AR-SMA mixtures with GPDa is higher than that of traditional mixtures.

## 1. Introduction

Crumb rubber, as a recycled material applied in asphalt, has gained more and more attention in recent years due to environmental benefits and property improvements in the virgin asphalt binder [1,2,3]. Asphalt rubber, the blend of virgin asphalt binder and crumb rubber, has excellent physical properties [4]. Moreover, there are generally many benefits using asphalt rubber mixtures: excellent resistance to cracking, improved durability, less road maintenance, lower road noise, etc. [5].

Various types of asphalt pavements have been used to study noise over the years, including Permeable European Mixture (PEM), Stone Matrix Asphalt (SMA), Asphalt Rubber Open-Graded Friction Course (AR-ACFC), Polymer Modified Open-Graded Friction Course (P-ACFC), ADOT’s Standard Open-Graded Friction Course (ACFC). The findings showed that the noise of AR-ACFC was lowest for all years and had a minimum increase in service years [6]. Bending beam test was selected to evaluate the fatigue performance of mixtures from different field sections in Arizona. The result illustrated that asphalt rubber mixture with gap-graded gradations had longer fatigue life than dense-graded mixes [7]. Way et al. [8] investigated the fatigue performance of numerous mixtures placed on various test sections in Arizona, including asphalt-rubber gap graded (ARAC), asphalt-rubber open-graded (ARFC), conventional dense graded, SMA, and conventional open-graded. Four point bending beam tests performed on these mixtures indicated that ARAC showed better resistance to fatigue cracking in comparison with the conventional open-graded mixture. However, the high-temperature performance of asphalt rubber mixtures did not perform well. Huang [9] investigated laboratory and field performance of mixtures from eight test sections in Louisiana. Gap-graded crumb-rubber modifier (CRM) mixtures had lower Marshall Stabilities and higher Marshall Flows in comparison with control conventional dense-graded mixtures. The CRM pavement sections showed similar or lower International Roughness Index than the control pavement with five to seven years of service. Santayana [10] examined the high-temperature performance of asphalt rubber binder and its mixtures. Multiple Stress Creep Recovery tests were carried to evaluate the high-temperature performance of asphalt rubber binder, and Flow Number and Wheel-Tracking tests were performed to investigate the high-temperature performance of asphalt rubber mixture. The findings showed conflicting results between the binder and its mixture testing. In particular, the high-temperature performance of rubberized mixtures was lower than that of dense-graded mixtures. Therefore, the high-temperature performance of asphalt rubber mixture still needed to be improved.

Many studies had demonstrated that it was an effective way to improve high-temperature performance by applying additives into asphalt mixtures [11,12,13]. The most common additives include Styrene–Butadiene–Styrene (SBS) and anti-rutting agent. This paper selected SBS and Granular Polymer Durable additive (GPDa) to analyze their effect on the design and performance of asphalt rubber mixtures. GPDa, a kind of anti-rutting agent, was found to perform well in enhancing the high-temperature performance of asphalt mixtures [14]. Additionally, it had been demonstrated that gap- and open-graded gradations were more suited to produce asphalt rubber mixtures than dense-graded gradations [15,16,17]. Hence, the SMA mixtures widely used in China were selected in this paper.

This paper aims to study the effect of additives on the laboratory performance of asphalt rubber Stone Matrix Asphalt (AR-SMA) with additives. The high-temperature and fatigue resistance of AR-SMA with additives (no additive, SBS, and GPDa) were studied by wheel tracking test and fatigue test, respectively; as well as the asphalt contents were considered. Three groups of AR-SMA: no additives, SBS, and GPDa were included, with no additives as a control group. Each group was investigated at three asphalt rubber content (ARC): 6.4%, 6.9%, 7.4% with regard to high-temperature and fatigue properties.

## 2. Materials and Methods 

### 2.1. Asphalt Binder

ESSO A-90 asphalt binder was used in this study. The Corbett test was conducted to evaluate the chemical composition of ESSO A-90. The results show that the percentages of Saturates, Aromatics, Resins, and Asphaltenes are 12.86, 56.49, 22.07, and 8.58, respectively. The physical properties of the virgin binder were measured in the laboratory according to Chinese National Standards [18], and are shown in Table 1.

### 2.2. Aggregate

Aggregates used in this paper were gneiss and separated by different sizes, including 10–15 mm, 5–10 mm, 3–5 mm, and 0–3 mm. Aggregates with the size larger than 3 mm are coarse aggregate, and aggregates with the size smaller than 3 mm are fine aggregates. Moreover, fine aggregates were produced by gneiss through a specific machine. The mineral filler was ground limestone. Table 2, Table 3 and Table 4 show the physical properties of coarse aggregate, fine aggregate, and mineral filler, respectively. The gradation of aggregates is shown in Table 5.

### 2.3. Crumb Rubber

The crumb rubber produced in Shaanxi (Shaanxi Chang-da-hua-chu Engineering Materials Technology Co., Ltd., Xi’an, China) was selected in this research. Crumb rubber obtained from scrap tires was produced by ambient grinding. The gradation of crumb rubber is shown in Table 6.

### 2.4. Additives

**Figure 1:**
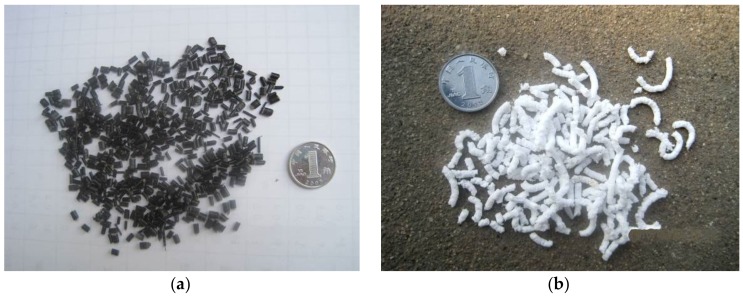
Additives: (**a**) Granular Polymer Durable additive (GPDa); (**b**) Styrene–Butadiene–Styrene (SBS).

In order to improve the mixture performance, SBS and GPDa were selected to produce asphalt rubber mixtures. They are shown in Figure 1. SBS is a common additive for improving the performance of asphalt mixtures, such as high-temperature and low-temperature performance. The SBS used in this paper was a star-type structure, and its properties are shown in Table 7. SBS (2%, by the weight of asphalt) was first used to prepare composite modified asphalt with SBS and crumb rubber, and then its mixture was produced. GPDa, one kind of anti-rutting agent, is produced in Xi’an, which can significantly increase the modulus of the mixture, resulting in improved high-temperature performance. Table 8 shows the main features of GPDa. GPDa (3‰, by the weight of the mixture) was added to asphalt rubber mixture.

### 2.5. Asphalt Rubber Preparation 

Asphalt rubber was produced in the laboratory by an open blade mixer (manufactured by Xi’an Ya-xing Engineering, Instrument Company, Xi’an, China) at a blending speed of 1000 rpm and a blending temperature of 180 °C–190 °C for 1 h. ESSO A-90 was modified with four contents of crumb rubber: 15%, 18%, 21%, and 24% (by the weight of asphalt rubber (weight of the virgin asphalt and crumb rubber)). The results showed that asphalt rubber with 18% crumb rubber performed best [19]. Therefore, 18% crumb rubber by the weight of virgin asphalt binder was selected in this paper. 

Table 9 shows the physical properties of asphalt rubber with 18% crumb rubber.

### 2.6. Specimen Preparation

Just like the common asphalt mixture, specimens were produced with a Wheel Compaction Device (Xi’an Ya-xing Engineering, Instrument Company, Xi’an, China) according to the specification [18]. Firstly, asphalt rubber was mixed with aggregates thoroughly at 170 °C–180 °C. Secondly, the mineral filler was added into for the following blending work. Thirdly, mixtures were compacted under the wheel at 160 °C–170 °C to obtain a slab specimen with the dimensions of 300 mm (length) × 300 mm (width) × 50 mm (height). The slab specimens were prepared for the wheel tracking test. 

In the fatigue test, the slab specimens were cut into several beams with a size of 250 mm (length) × 30 mm (width) × 35mm (height). Three specimens were prepared for both the wheel tracking and fatigue test.

### 2.7. Mechanical Property Testing

The wheel tracking and fatigue tests were used to evaluate the high-temperature performance and fatigue resistance of AR-SMA with different additives, respectively. Moreover, the asphalt contents were considered. The testing procedures have complied with Chinese National Standard [18].

#### 2.7.1. Wheel Tracking Test

According to T0719 in the standard [18], a slab was tested at the temperature of 60 °C with a wheel-pressure of 0.7 MPa. The parameter of dynamic stability was employed to characterize the high-temperature stability of the tested mixture and was determined using Equation (1):

(1) $$DS=\frac{42\times 15}{d_{60}-d_{45}}$$

where $d_{60}$ is the rutting depth (mm) at 60 min, $d_{45}$ is the rutting depth at 45 min, 630 is the number of load cycles applied between 45 and 60 min.

A higher dynamic stability value usually indicates an excellent high-temperature performance (rutting resistance) of the mixture [20].

#### 2.7.2. Fatigue Test

The fatigue test can assess the cracking resistance of asphalt specimens under a repeated load. It is a stress-controlled test and performed using American Materials Testing and Simulation Machine (CMT5105, MTS, Eden Prairie, MN, USA) in accordance with the T0715 of the specification [18]. The specimens were tested at the temperature of 15 °C with the strain rate 50 mm/min and loading frequency 10 Hz. Fatigue failure occurs when the load reaches a maximum.

The fatigue resistance was evaluated according to the fatigue curves generated by testing, which introduces the relationship between fatigue strength and fatigue life. The fatigue equation in this study was described with the formula given in Equation (2):

(2) $$\mathrm{lg}N_{f}=n\times \mathrm{lg}\sigma +k$$

where $N_{f}$ is the fatigue life (in cycles); σ is the fatigue stress (MPa) applied during the test. The equation provides a linear relationship between them using a denary logarithm, in which *n* is the slope and *k* is the intercept. Fatigue resistance decreases with increasing values of *n*. Conversely, fatigue life increases with increasing values of *k* [20]. 

## 3. Results and Discussion

### 3.1. Mixture Design

The Marshall Mix design procedure was used for mixture design. AR-SMA with a nominal maximum size of 13.2 mm was selected in this study. Table 10 shows the gradation of AR-SMA.

According to the OAC of the traditional SMA, ARC 6.1% was selected as the initial value. Subsequently, ARC 6.1%, 6.4%, and 6.7% were used for mixtures design [5]. Five specimens were prepared for each ARC. The volumetric properties of mixtures with different ARC were analyzed. At last, the asphalt content of 6.4% was selected as the optimal value.

According to previous research results [21] and experiences, OAC of mixtures with additives may be 0.5% higher than those without additives. Therefore, three contents (6.4%, 6.9%, and 7.4%) were selected for mechanical property testing in this paper.

### 3.2. Mechanical Property Testing

#### 3.2.1. Wheel Tracking Test

Three groups of AR-SMA, with no additives, SBS, and GPDa, were prepared for the test at three ARC, 6.4%, 6.9%, and 7.4%. Then, their high-temperature performances were evaluated.

**Figure 2:**
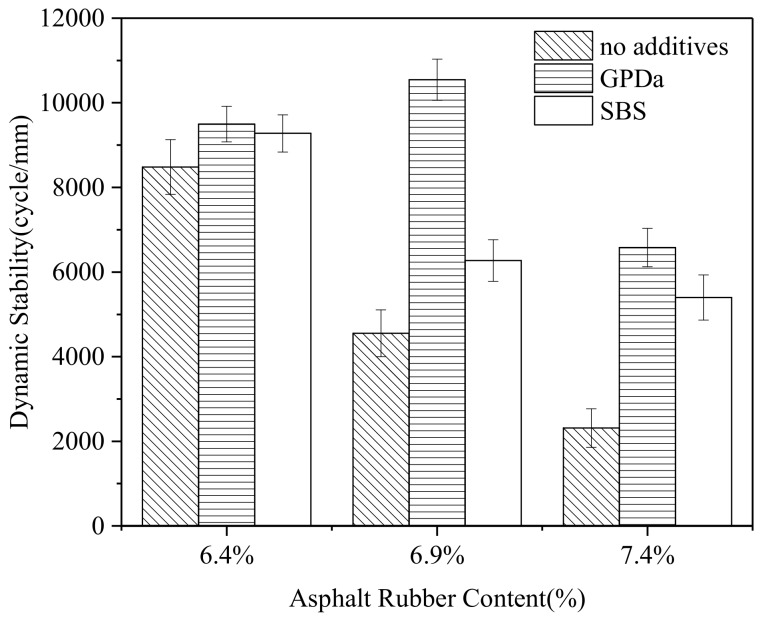
Dynamic stability of mixtures with different asphalt rubber contents (ARCs) and different additives. GPDa: Granular Polymer Durable additive; SBS: Styrene–Butadiene–Styrene.

Figure 2 shows the wheel tracking test results for mixtures with different ARC and different additives.

##### Different ARC

As seen in Figure 2, for mixtures with no additives, when the ARC is 6.4%, the dynamic stability is about more than 8000 cycle/mm, more or less equal to that of traditional SMA. Subsequently, with asphalt rubber content increased to 6.9% and 7.4%, the dynamic stability of the mixture decreases sharply. The reason is mainly that too much asphalt rubber is more likely to cause deformation of the mixture under the load [22,23].

For mixtures with GPDa, the dynamic stability of AR-SMA first increases and then decreases with increasing ARC. This may be explained as follows: with low ARC, the GPDa could not be saturated completely by asphalt rubber. So, its advantages, increasing stiffness, the viscosity of asphalt binder, or the asphalt-aggregate adhesion, could not perform very well [24]. As the ARC grows, the function of the additive becomes more and more active, and the performance is improved consequently.

For mixtures with SBS, the dynamic stability of AR-SMA has the same trend as that of mixtures with no additives. 

##### Different Additives

In Figure 2, when the ARC is 6.4%, the dynamic stability of mixtures with additives is a little larger than that with no additives. Meanwhile, GPDa and SBS have a similar level. 

Then, the performance of mixture with GPDa is improved by 2.31 times, and for SBS, it is 1.47 times, compared to mixtures with no additives and ARC of 6.9%. It indicates that GPDa can provide better high-temperature performance than SBS. 

When the ARC is 7.4%, the performance of the mixture is increased by 2.71 times and 2.27 times with GPDa and SBS, respectively. 

Figure 2 shows that mixture with GPDa has the best high-temperature performance at 6.9% among all mixtures. Just as mentioned above, the result may be due to the fact that 6.9% may be the optimal ARC, which makes the additive perform very well.

##### The Significance of Additives and ARC

Table 11 is the result of the statistical significance of additives and ARCs on the high-temperature performance, according to two-way analysis of variance (ANOVA). It can be seen that for additives, the F value is less than F_critical_, and for ARC, the F value is larger. The result indicates that, compared with additives, ARC has a more significant effect on the high-temperature performance of mixture at the 95% confidence level.

The results indicate that the optimum asphalt content is very important for the design and performance of the mixture. Similarly, the additive may be selected at the appropriate asphalt content in order to perform well.

#### 3.2.2. Fatigue Test

In the wheel tracking test, mixture with GPDa shows a better performance than the one with SBS. So, two groups of mixtures with no additives and GPDa were prepared at three ARCs, 6.4%, 6.9%, and 7.4%. Fatigue curves were plotted and analyzed.

**Figure 3:**
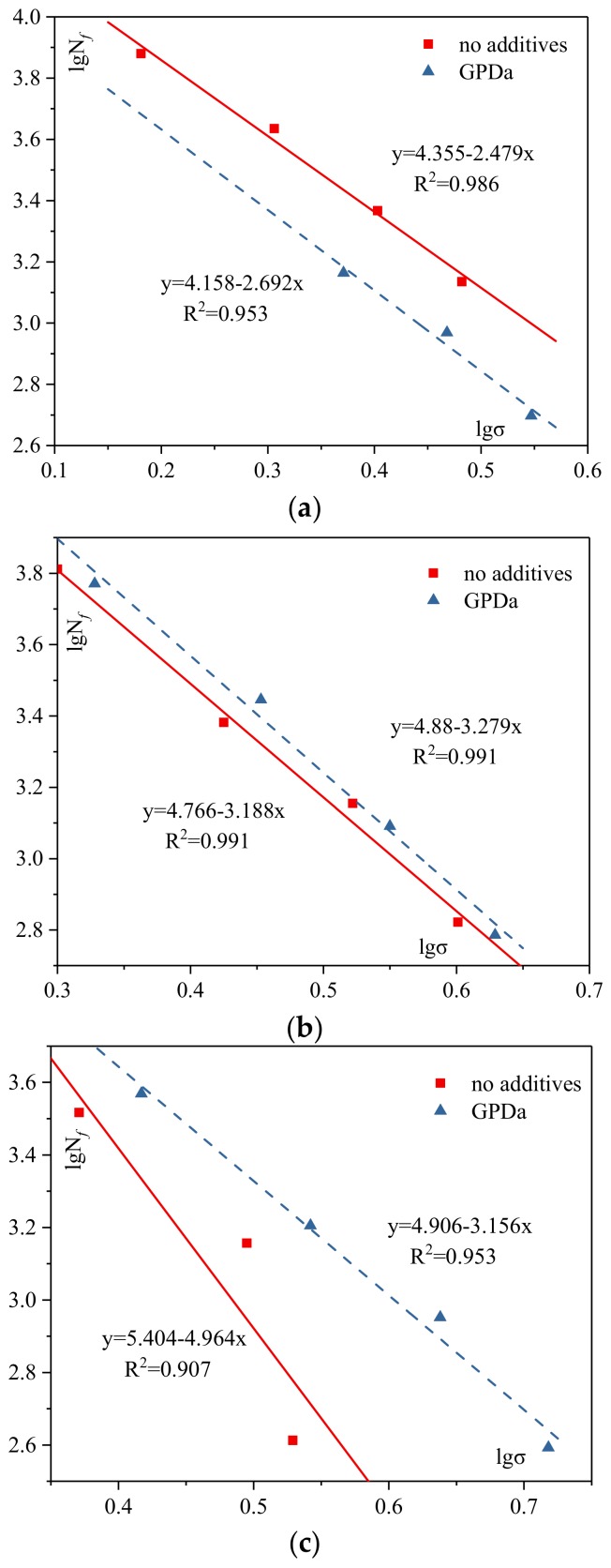
Curves for mixtures at different asphalt rubber contents (ARCs): (**a**) ARC of 6.4%; (**b**) ARC of 6.9%; (**c**) ARC of 7.4%. GPDa: Granular Polymer Durable additive.

**Figure 4:**
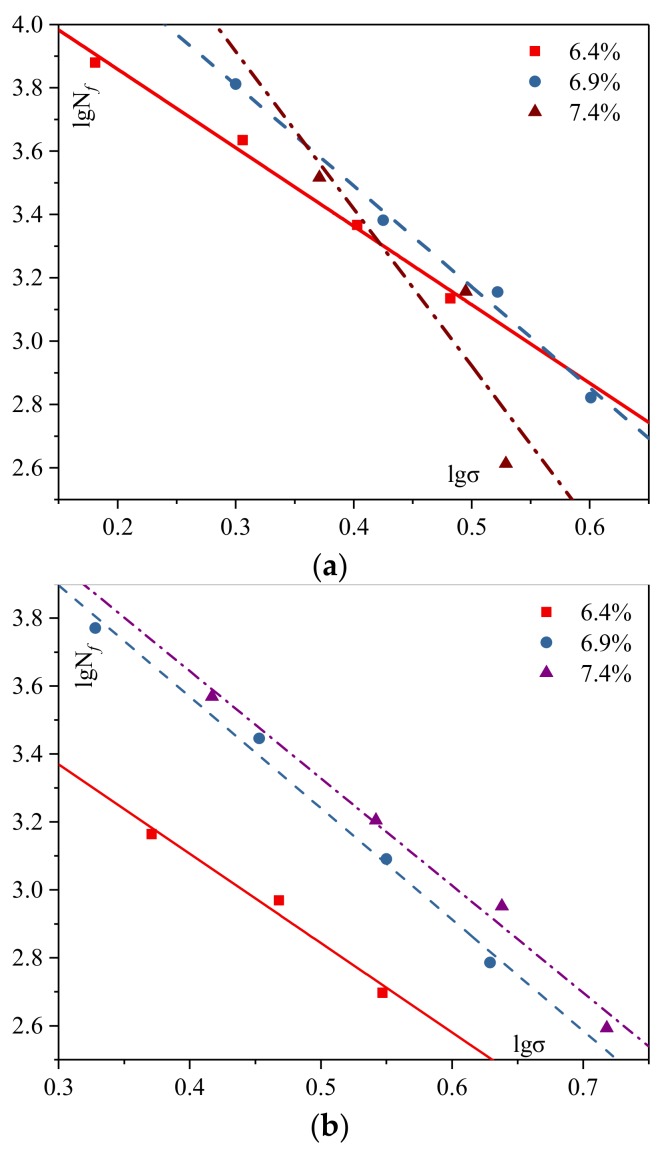
Curves for mixtures with different additives: (**a**) mixtures with no additives; (**b**) mixtures with Granular Polymer Durable additive (GPDa).

The fatigue test results for mixtures with different additives and different ARCs are presented in Figure 3 and Figure 4, separately.

##### Different Additives

In Figure 3a, it can be seen that n value of mixture with no additives is higher than that of the mixture with GPDa, while k value is lower. The results indicate that using GPDa reduces fatigue life and resistance to fatigue cracking. It is mainly because unsuitable asphalt content makes GPDa not perform well. However, the difference between these two parameters is not obvious, indicating that using GPDa has a slight impact on the fatigue performance of mixtures.

Being significantly different from Figure 3a, Figure 3b shows that both k and n values of AR-SMA with GPDa are greater than mixtures with no additives. It is mainly because asphalt-rubber mixture with GPDa has higher stiffness, resulting in higher fatigue life but lower sensitivity to fatigue cracking. Simultaneously, the difference between these two parameters is smaller with increasing ARC. It reveals that the difference in fatigue performance is smaller with increasing ARC. Moreover, both k and n values of AR-SMA with or without additives increase with increasing ARC. It means that both fatigue life and sensitivity to fatigue cracking increase with increasing ARC.

Figure 3c shows that n value of mixture with GPDa is lower than that without additives, but k value is higher. Simultaneously, the difference between these two parameters in different groups becomes higher. Moreover, data is farther away from the regression line for mixtures with no additives at ARC 7.4%. It means that excessive asphalt content generates test error.

##### Different ARC

As shown in Figure 4a, with the increasing asphalt rubber contents, k value becomes larger, which indicates a better fatigue life for mixtures with no additives. In Figure 4b, the tendency is the same for the mixture with GPDa: when the ARC grows, k value extends, and the performance is also raised. This can be explained as follows: more ARC will result in thicker asphalt films on the aggregates’ surface, which contributes to the adhesion between asphalt and aggregate, as well as the crack resistance of mixtures [25,26]. Moreover, with increasing ARC, the fatigue performance of AR-SMA with GPDa changes slower than mixture without additive, indicating that using GPDa reduces sensitivity to fatigue performance with various ARC.

##### The Significance of Additives and ARC

Two-way ANOVA was carried out for statistical analysis of fatigue performance of AR-SMA with different additives and ARCs at 95% confidence level. The results are shown in Table 12. Various additives and ARC significantly affect stress in statistics but have no significant effect on fatigue life in statistics.

## 4. Conclusions

This paper describes a study of the performance of asphalt rubber mixture with additives. From the results and analysis, the following conclusions can be drawn:

- In the wheel tracking test, both SBS and GPDa could improve the high-temperature performance of the mixture. The rutting resistance of AR-SMA with GPDa at ARC 6.9% performs best.
- GPDa is an alternative additive to improve the high-temperature performance of mixtures.
- Under the condition of mixtures with appropriate ARC, AR-SMA with GPDa has higher fatigue life and sensitivity to fatigue cracking than that without additives.
- Both ARC and additives significantly affect rutting performance statistically, and ARC has a greater impact on rutting performance than additives. The variance analysis of the fatigue test shows that various additives and ARC significantly affect stress statistically, but have no significant effect on fatigue life.
- Optimal asphalt content is very important for the design and performance of mixture. Similarly, the additive may be selected at the appropriate asphalt content in order to perform well.
- Further study should be undertaken on the effect of additives on other performances of mixtures, for example, moisture susceptibility, aging, and low-temperature performance.

## Figures and Tables

**Table 1:** Physical Properties of Asphalt Binder.

Test Items	Unit	Value	Specification
25 °C penetration	0.1 mm	81	T0604
10 °C ductility	cm	72	T0605
Softening point	°C	47.5	T0606

**Table 2:** Physical Properties of Coarse Aggregate.

Test Items	Unit	Value			Specification
		10–15 mm	5–10 mm	3–5 mm	
Crushing value	%	13.4	-	-	T0316
Los Angeles abrasion	%	11.8	-	-	T0317
Apparent relative density	-	2.856	2.857	2.812	T0304
Bulk relative density	-	2.781	2.762	2.709	
Water absorption	%	0.51	0.63	0.59	
Flat or elongated	%	4.0	6.7	-	T0312

**Table 3:** Physical Properties of Fine Aggregate.

Test Items	Unit	Value	Specification
Apparent relative density	-	2.797	T0328
Mud content (percent of <0.075 mm)	%	1.1	T0333
Sand equivalent	%	95.5	T0334
Angularity	s	57.1	T0344

**Table 4:** Physical Properties of Mineral Filler.

Test Items	Unit	Value	Specification
Apparent relative density	-	2.799	T0352
Water absorption	%	0.2	
Grain sizes <0.6 mm	%	100.0	T0351
<0.15 mm	%	95.0	
<0.075 mm	%	90.1	
Hydrophilic coefficient	-	0.60	T0353

**Table 5:** The Gradation of Aggregates.

Aggregates with Different Sizes	Passing Percentage of Aggregates with Different Sizes/%									
	16	13.2	9.5	4.75	2.36	1.18	0.6	0.3	0.15	0.075
10–15 mm	100	92.3	6.1	0.1	0.1	0.1	0.1	0.1	0.1	0.1
5–10 mm	-	100	96.1	1.5	0.2	0.2	0.2	0.2	0.2	0.2
3–5 mm	-	-	100	88.1	4.3	1.1	1.1	1.1	1.1	1.1
0–3 mm	-	-	-	100	92.0	4.4	1.3	1.3	1.3	1.3
Mineral filler	-	-	-	-	100	100	100	99	97	89

**Table 6:** The Gradation of Crumb Rubber.

**Sieve Sizes (mm)**	1.18	0.6	0.3	0.15	0.075
**Passing Percentage/%**	100.0	99.2	66.6	25.4	5.2

**Table 7:** Physical Properties of Styrene–Butadiene–Styrene (SBS).

Test Item	Rate (S/B)	Rate of Liquid Volume/%	Volatile ≤%	Ash Content ≥%	Tensile Strength ≥MPa	Shore Hardness A	Melt Flow Rate g/min
Value	30/70	0	0.7	0.2	8	70	0~1

**Table 8:** Main Features of Granular Polymer Durable additive (GPDa).

Test Item	Density/(g/cm^3^)	Melting Point/°C	Tensile Strength/MPa	Elongation at Break/%	Melt Flow Rate/(g/10 min)	Ash Content/%
Value	0.94	146	22.0	8.7	7.431	2.4

**Table 9:** Physical Properties of Asphalt Rubber.

Asphalt Binder	Crumb Rubber	180 °C Viscosity (Pa·s)	25 °C Penetration (0.1 mm)	Softening Point (°C)	5 °C Ductility (cm)	Elastic Recovery (%)
ESSO A-90	Shanxi	3.650	63	59.4	17.4	53

**Table 10:** Gradation for asphalt rubber Stone Matrix Asphalt (AR-SMA).

**Sieve Sizes (mm)**	16	13.2	9.5	4.75	2.36	1.18	0.6	0.3	0.15	0.075
**Passing Percentage (%)**	100	97.9	63.5	27	23.5	20	16.4	12.9	9.7	6

**Table 11:** ANOVA Results for Dynamic Stability.

Source of Variation	Sum of Square	Degree of Freedom	Mean of Square	F	*p*-Value	F_critical_
Additives	2.1 × 10^7^	2	1.0 × 10^7^	5.57	0.070	6.94
ARC	2.9 × 10^7^	2	1.4 × 10^7^	7.86	0.041	6.94
Error	0.057	4	0.011	-	-	-
Total	18.123	11	-	-	-	-

**Table 12:** ANOVA Results for Fatigue Test.

Source of Variation		Degree of Freedom	Mean of Square	F	*p*-Value
Additives	lgσ	1	0.052	20.702	0.000
	lgN_f_	1	0.349	3.436	0.085
ARC	lgσ	2	0.036	7.036	0.008
	lgN_f_	2	0.583	2.874	0.090
